# Chinese Medicine for Treating Endocrinology-Related Disease

**DOI:** 10.1155/2015/680290

**Published:** 2015-03-26

**Authors:** Yizhou Xin, Ying Zhang, Chunchao Han

**Affiliations:** ^1^The Affiliated Hospital of Shandong University of Traditional Chinese Medicine, Jinan 250011, China; ^2^Shandong Eye Hospital, Jinan 250021, China; ^3^School of Pharmacy, Shandong University of Traditional Chinese Medicine, Jinan 250355, China

According to new research accepted for publication in the Endocrine Society's Journal of Clinical Endocrinology & Metabolism, Chinese herbal medicines are well recognized for their medicinal properties and have been used in traditional medicine for millennia. The medicinal effects attributed to herbal medicines, based mainly on uncharacterized substances or extracts, include depression management, cerebrovascular disease management, Alzheimer's disease management, and antianxiety, immunomodulatory, antioxidant, radical scavenging, anti-inflammatory, antihyperlipidemic or antihypercholesterolemic, hepatoprotective, and antidiabetic effects.

4-Hydroxychalcone (4HCH) is an alpha, beta-unsaturated ketone with the core structure of chalcone and one hydroxyl-substituent on the 4-position of the A ring. It belongs to the largest class of plant secondary metabolites and is considered to be precursor of flavonoids and isoflavonoids serving plant defense mechanisms to counteract reactive oxygen species in order to survive and prevent molecular damage and damage by microorganisms, insects, and animals. F. Zhang investigated the preventive effects of 4-hydroxychalcone (4HCH) on resistant hypertension. The cryptochrome-null mice, which received high-salt treatment, were treated orally with 4HCH 10 mg/kg, 4HCH 20 mg/kg, and 4HCH 40 mg/kg, respectively. The salt administration in cryptochrome-null mice is able to induce an increase in systolic pressure which is associated with hyperaldosteronism, inflammation, and kidney injury. Treatment with 40 mg/kg 4HCH reduced systolic hypertension, serum IL-1*β*, and TNF-*α* levels and suppressed the activation of nuclear factor kappa-light-chain-enhancer of activated B cells (NF-*κ*B) and renal injury. The impact of 4HCH on the hyperaldosteronism, inflammation, and kidney injury provides new insights for future development of therapeutic strategies in resistant hypertension.

*Agaricus blazei *Murrill (ABM), an edible mushroom is native to Brazil, which is widely used for nonprescribed and medicinal purposes. C. Han investigated the medium composition of ABM that was optimized using response surface methodology for maximum mycelial biomass and extracellular polysaccharide (EPS) production. The model predicts to gain a maximal mycelial biomass and extracellular polysaccharide at 1.047 g/100 mL and 0.367 g/100 mL, respectively, when the potato is 29.88 g/100 mL, the glucose is 1.01 g/100 mL, and the bran is 1.02 g/100 mL. The verified experiments showed that the model was significantly consistent with the model prediction and that the trends of mycelial biomass and extracellular polysaccharide were predicted by artificial neural network. After that, the optimized medium was used for the submerged culture of ABM. Then, alcohol-induced liver injury in mice model was used to examine the protective effect of ABM cultured using the optimized medium on the liver. And the hepatic histopathological observations showed that ABM had a relatively significant role in mice model, which had alcoholic liver damage. Y. Gao investigated the effect of* Agaricus brasiliensis *extract (ABE) on phenylhydrazine-induced neonatal jaundice in rats. Administration of ABE dose-dependently reduced the elevated bilirubin level induced by phenylhydrazine. It can be somewhat supported from the results of* in vitro *bilirubin degradation experiment. ABE treatment also reduced the total antioxidant status (TAOS), cascade O_2_^−^/SOD, level of NF-*κ*B protein, and adrenomedullin (AM). Overall, the results of this study demonstrated that* Agaricus brasiliensis *extract may be beneficial to reducing bilirubin level without causing hepatotoxicity in neonatal jaundice.

Crocetin (C_20_H_24_O_4_) is one of the major active constituents of saffron, which is derived from the dried stigma of* Crocus sativus* L., belonging to the Iridaceae family. C.-D. Li investigated the effect of crocetin (C_20_H_24_O_4_) on methylcholanthrene- (MCA-) induced uterine cervical cancer in mice. After the mice were treated orally with crocetin, maleic dialdehyde (MDA), polymorphonuclear cells (PMN), interleukin-1*β* (IL-1*β*), and tumor necrosis factor-*α* (TNF-*α*) were examined by ELISA or immunohistochemistry. The inducible nitric oxide synthase (iNOS) activation in* Hela *cells was analyzed using fluorescence microscopy for light microscopic examination. The MCA-mice showed a significant increase in plasma MDA, PMN, IL-1*β*, TNF-*α*, and nitrates levels. At the same time, the mRNA level of COX-2 in* Hela *cells was also significantly increased. These changes were attenuated by crocetin supplementation in the MCA mice. Crocetin supplementation in the MCA mice also showed the protection against cervical cancer. These results suggest that crocetin may act as a chemopreventive and an anti-inflammatory agent.

*Fructus Ligustri Lucidi *(FLL) is a well-known invigorator in Chinese materia medica with the effects of hepatoprotective effect, anticancer activity, antioxidant activity, and so on. C. Han reviewed the pharmacological effects of* Fructus Ligustri Lucidi.* It contains a number of bioactive components. Moreover, FLL has been known to have hepatoprotective effect, anticancer activity, antioxidant activity, immunomodulating effect, antiviral activity, and antiosteoporosis activity and other pharmacodynamic effects have been demonstrated as well, such as the treatment of coronary heart disease. The chemical constituents of FLL include polysaccharides, triterpenes, secoiridoids, and flavonoids, which maybe contributed to the pharmacological activities of FLL. Therefore, the pharmacological effects and health function of* Fructus Ligustri Lucidi *are more and more focused on in the world.

Breviscapine is an active ingredient of flavonoids extracted from dried* Erigeron breviscapus* (Vant.) Hand-Mazz. Z. Zhou reviewed combined therapy of diabetic peripheral neuropathy with breviscapine and mecobalamine. A meta-analysis on combined therapy of diabetic peripheral neuropathy (DPN) with breviscapine and mecobalamine was performed to evaluate the efficacy of this therapy. A total of 17 articles including 1398 DPN patients were identified. Homogeneity was observed among different studies (*P* = 0.74). The efficacy of combined therapy with breviscapine and mecobalamine was significantly better than that in control group (*P* < 0.0001 (OR = 5.01, 95% CI: 3.70–6.78)). Available findings suggest that the therapeutic efficacy of breviscapine combined with mecobalamine is superior to mecobalamine alone, and this strategy is required to be popularized in clinical practice.

*Cordyceps *is a genus of the family Clavicipitaceae that has been used in traditional oriental medicine for centuries. Recent studies have demonstrated that the bioactive components isolated from this genus have various pharmacological actions. Among them, cordycepin, also known as 3-deoxyadenosine, has been shown to possess multiple pharmacological activities such as inhibition of tumour growth, modulation of the immune response, and suppression of reactive oxygen species. G.-Y. Zhao reported that cordycepin can act as anti-inflammatory agent in magnesium silicate-induced inflammation in osteoporosis. The beneficial effects of cordycepin on improvement of osteoporosis in rats were attributable mainly to decrease ALP activity, TRAP activity, and CTX level. At the same time, cordycepin also increases the OC level in ovariectomized osteopenic rats. The histological examination clearly showed that dietary cordycepin can prevent bone loss caused by estrogen deficiency. These experimental results suggest that complement cordycepin is protective after ovariectomized osteopenic in specific way.

*Yizhou Xin* *Ying Zhang* *Chunchao Han*

